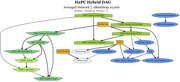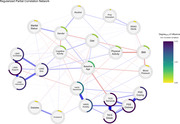# Intervening paths: A dual networks approach to modifiable dementia risk in healthy older adults

**DOI:** 10.1002/alz.092146

**Published:** 2025-01-09

**Authors:** James J R Brady, Larissa Bartlett, Aidan Bindoff, Kimberley Norris, Eddy Roccati, James C Vickers, Duncan Sinclair

**Affiliations:** ^1^ University of Tasmania, Hobart, TAS Australia

## Abstract

**Background:**

Multi‐domain initiatives which target modifiable, lifestyle‐associated, dementia risk factors are promising tools for dementia prevention. However, those at greatest risk of preventable dementia likely have the least capacity to enact change. Interventions may improve outcomes for those most vulnerable by looking up‐stream. Doing so may identify targets of intervention which influence modifiable dementia risks. Applying an emerging method, we reveal candidates for targeted intervention using partial correlation and direct‐path machine‐learned networks.

**Method:**

Observations from the Island Study Linking Ageing and Neurodegenerative Disease (ISLAND) Resilience Initiative (*n* = 1225) were sourced from the longitudinal ISLAND Study of adults over 50. Regularized partial correlation network (RPCN) was plotted with demographic and dementia risk factors using glasso and extended Bayesian Information Criteria. Removing missingness, we systematically selected nonparanormal data transformation and a H2PN algorithm to bootstrap 10,000 Bayesian networks. Our directed acyclic graph (DAG) comprised connections averaged across at least 85 per‐cent of the bootstraps. DAG network stability was tested by incrementally diminishing number of observations. Common, highly connected, and intervenable variables which led to most‐influenced modifiable dementia risks were identified by contrasting the DAG and RPCN.

**Result:**

RPCN showed modifiable risk domains of depression, cognitive activity, and MIND diet adherence were most influenced by partially correlated factors. As revealed by the DAG, pathways leading to depression included a direct link from social support availability, and indirect links from age through anxiety and the stress variables, excepting PSS’s unable to cope subscale. Gender and social subscales, except interaction frequency, led to cognitive activity, and dietary adherence (through cognitive activity and BMI). Of the up‐stream variables, identified links between stress, anxiety, and depression were the strongest, visible as thicker connections in the RPCN.

**Conclusion:**

We present candidates for focused intervention, demonstrating utility of an emerging dual‐network technique. We unveil a complex RPCN network, highlighting modifiable dementia risk factors most influenced by correlated variables. Combining the DAG’s machine‐learned pathways with the RPCN, we provide support for targeting symptoms of stress and anxiety, and fostering improved social connectivity and quality, to address dementia risk associated with levels of depression, cognitive activity, and MIND diet adherence.